# The Impact of Prepartum Platelet Count on Postpartum Blood Loss and Its Association with Coagulation Factor XIII Activity

**DOI:** 10.1159/000529020

**Published:** 2023-01-18

**Authors:** Romana Brun, Torsten Hothorn, Eva Eigenmann, Marie Louise Frevert, Roland Zimmermann, Wolfgang Korte, Christian Haslinger

**Affiliations:** ^a^Department of Obstetrics, University Hospital of Zurich, Zurich, Switzerland; ^b^Epidemiology, Biostatistics and Prevention Institute, University of Zurich, Zurich, Switzerland; ^c^University of Zurich, Zurich, Switzerland; ^d^Center for Laboratory Medicine, Hemostasis and Hemophilia Center, St. Gallen, Switzerland

**Keywords:** Postpartum hemorrhage, Thrombocytopenia, Factor XIII, Platelet transfusion, Platelets

## Abstract

**Background:**

Postpartum hemorrhage is a leading cause of maternal morbidity and mortality worldwide. Contradictory information exists regarding the relevance of prepartum platelet count on postpartum hemorrhage. We have shown prepartum coagulation factor XIII to be associated with postpartum blood loss; however, little is known about the association of platelet count with factor XIII activity. Our objectives were, first, to evaluate the impact of prepartum platelet count on measured postpartum blood loss in the context of prepartum measurements of coagulation factors I, II, and XIII and, second, to evaluate the association of platelet count with coagulation factor XIII, both pre- and postpartum.

**Material and Methods:**

This is a secondary analysis of a prospective cohort study (PPH 1,300 study) which analyzed the impact of prepartum blood coagulation factors on postpartum blood loss in 1,300 women. Blood loss was quantified using a validated technique. The impact of prepartum platelet count on measured blood loss was assessed by continuous outcome logistic regression; the association of platelet count with factor XIII activity by Spearman rank correlation.

**Results:**

Prepartum platelet count was significantly associated with measured postpartum blood loss: every one unit (G/L) increase in prepartum thrombocytes was associated with an odds ratio of 1.002 (95% confidence interval, 1.001−1.004, *p* = 0.005) to keep blood loss below any given cut-off level. This means that the probability of postpartum hemorrhage decreases with increasing prepartum platelet levels. Moreover, a significant association of platelet count with factor XIII activity was shown (Spearman rank correlation coefficient for prepartum values 0.228, *p* < 0.001, and for postpartum values 0.293, *p* < 0.001).

**Discussion/Conclusion:**

The significant association of prepartum platelet count and postpartum blood loss as well as the association of platelet count with blood coagulation factor XIII activity support the likely role of platelets in preventing postpartum hemorrhage and support the new guidelines for the treatment of postpartum hemorrhage in Germany, Austria, and Switzerland, which calls for optimizing platelet counts peripartally in case of postpartum hemorrhage. A possible effect of platelets on the level of circulating factor XIII cannot be ruled out and should prompt further investigation.

## Introduction

Thrombocytopenia in pregnancy is frequently encountered by obstetricians or hematologists. Its prevalence in all pregnancies is estimated to be 10% [1]. Little and contradictory evidence exists on the role of prepartum platelet counts on postpartum blood loss and its possible influence on the other coagulation factors. Over the last years, evidence that platelet counts have an influence on the volume of postpartum hemorrhage has been accumulating; however, most studies interpreted the results by categorization, using thresholds of platelet counts (e.g., <100 G/L or <150 G/L) instead of interpreting distributed data continuously, thus reflecting the biological reality more adequately. Several studies using the threshold approach demonstrate that mild thrombocytopenia (100−149 G/L) increases the likelihood of postpartum hemorrhage in cesarean deliveries as well as in vaginal deliveries [2, 3, 4, 5].

On the contrary, some other studies did not reveal any correlation of platelet count with an increased risk for postpartum hemorrhage [6, 7, 8]. However, some of these mentioned studies might have been underpowered by including fewer than 200 parturients [6, 7].

The Association of the Scientific Medical Societies (AWMF) recently updated its guideline on the management of postpartum hemorrhage [9]. An expert group of obstetricians, anesthesiologists, and hematologists from Germany, Switzerland, and Austria were part of the AWMF scientific panel developing the revised guideline.

The algorithm on hemostatic therapy for PPH in the updated guideline recommends 6 steps to optimize therapy (temperature regulation, pH correction, early use of tranexamic acid, fibrinogen, and blood coagulation factor X III [“FXIII”] substitution) in an escalating protocol. The sixth step, which was newly added to the guideline, suggests platelet transfusions for continued bleeding that requires blood product support with the aim of reaching a platelet count >100 G/L [10, 11].

Recently, the studies of Haslinger [12] and Bamberg [13] showed that prepartum FXIII levels are associated with postpartum blood loss. The influence of platelets on FXIII activity has been discussed in a basic research environment [14] but not in a clinical setting. Available data show that the catalytic A subunits of FXIII (FXIII-A) are present in the cytoplasm of platelets [14]. Moreover, activated FXIII is likely involved in certain phases of the platelet spreading process and might participate in clot retraction, as was shown in a mouse model (10).

With the implementation of the new AWMF guidelines for the management of postpartum hemorrhage, platelet transfusion is gaining further importance. Due to the scant evidence available on this subject, our aims were, first, to evaluate the role of platelets on postpartum blood loss in a continuous logistic regression analysis rather than the categorical analysis used so far and, second, to evaluate the association between platelet numbers and FXIII activity.

## Methods

This secondary analysis of a prospective single-center cohort study [12] was performed at the Department of Obstetrics, University Hospital Zurich, Switzerland, between October 2015 and November 2016 following Institutional Review Board approval (KEK-ZH 2015-0011, ClinicalTrials.gov registration NCT02604602). The study was funded by University Hospital Zurich, CSL Behring Switzerland (unrestricted research grant), the Center for Laboratory Medicine St. Gallen, and a private donor (a former patient). The nonacademic funding entities involved (i.e., CSL Behring and the private donor) had neither influence on the design or the performance of the study; nor on the collection, management, analysis, or interpretation of data; the preparation, review, or approval of the manuscript; or on the publication.

Prospective data collection was performed by dedicated research personnel, using patient data from the general and obstetrics-specific clinical information systems. Blood coagulation factors I, II, and XIII were analyzed at the Center for Laboratory Medicine, Hemostasis and Hemophilia Center, St. Gallen, Switzerland, only after the last patient was enrolled, thus preventing potential treatment bias. The samples for pre- and postpartum platelet counts were immediately analyzed at the University Hospital Zurich and were unblinded for the personnel.

Women admitted to the labor ward before vaginal delivery or cesarean section was included if they were ≥18 years of age and their pregnancy was ≥22 weeks of gestation. Informed written consent was obtained from all study participants before enrolment. Patients with known congenital disorders of hemostasis, on anticoagulant therapy, and women with preeclampsia or eclampsia were not eligible. Enrolment was consecutive.

The prepartum blood sample was drawn in the 36 h preceding the onset of delivery, defined as regular contractions or rupture of membranes, whichever occurred first. The postpartum blood sample was taken 24−48 h after delivery. Blood loss measurement followed a strict protocol, which has been validated and was published previously [15]: Immediately after the birth, the midwife places a fresh drape under the woman‧s pelvis to collect blood. The drape is regularly checked and if continued bleeding is observed, the drape is weighed on a neonatal balance which is installed in every suite. If the overall weight (i.e., fluid minus drape) exceeds 300 g, a plastic bag with a quantitative scale is placed under the pelvis for blood collection and exact measurement. Details of the study protocol, blood sample processing, blood loss measurement, and details of the statistical methods applied have been published before [12].

Fibrinogen concentration (Clauss assay) and FII activity (one-stage clotting assay) were determined on an ACL 500 coagulation analyzer (Instrumentation Laboratory) according to the manufacturer‧s recommendations; FXIII activity was measured by chromogenic assay (Berichrome) on an XP analyzer (Siemens), also according to the manufacturer‧s recommendations. Prepartum hemoglobin (low levels of which may influence postpartum blood loss) was analyzed on an ADVIA (Siemens) or XN-20 (Sysmex) instrument.

Platelets were analyzed on an ADVIA (Siemens) or XN-20 (Sysmex) instrument. According to the platelet values, one of the following methods was chosen: impedance measurement principle, fluorescence optical method, or fluorescence flow cytometry.

Baseline demographics, fetomaternal and perinatal characteristics, and prepartum coagulation factor values were stratified by mode of delivery and are presented in Table 1 using descriptive statistics. In a first step, the conditional distribution of measured blood loss (MBL) in relation to prepartum platelet count (G/L), hemoglobin (g/L), fibrinogen (g/L), FII (%), and FXIII (%) was estimated by continuous outcome logistic regression [16, 17].

In this model, all possible binary logistic regression models for all MBL volumes were estimated, thus allowing us to apply this model to any blood loss-cut-off point (i.e., the regression coefficients were treated as constants). This statistical approach and its validity are explained in detail elsewhere [12]. In summary, the regression coefficients describe the odds ratio and assess the change associated with a one-unit increase in one of the evaluated prepartum blood parameters at any given blood loss. Thus, the calculated odds ratios describe the effect of a one-unit increase in the respective prepartum parameter on the probability to remain below any amount of MBL chosen. A binary logistic regression model using the conventional cut-off at 500 mL, as postpartum hemorrhage is defined in vaginal deliveries [18], was performed as well, to compare the binary with the more complex continuous logistic regression model. The regression coefficients calculated by the continuous outcome logistic regression model and by the MBL cut-off dependent binary logistic regression model are depicted graphically. Moreover, a spearman rank correlation coefficient was calculated to evaluate the association of platelet count with Factor XIII activity both in the prepartum as well as the postpartum setting.

## Results

Prepartum platelet counts significantly influenced MBL: every one unit (G/L) increase in prepartum platelets was associated with an odds ratio of 1.002 (95% confidence interval, 1.001−1.004, *p* = 0.005) to keep blood loss below any given volume (continuous outcome logistic regression model). In other words, the probability of postpartum hemorrhage decreased with increasing prepartum platelet counts. After stratification for delivery mode, the effect observed in the continuous outcome logistic regression model remained significant for vaginal deliveries (OR 1.002, 95% CI 1.000−1.005, *p* = 0.05) and showed a similar trend for cesarean deliveries (OR 1.002, 95% CI 1.000−1.005, *p* = 0.08).

**Fig. 1:**
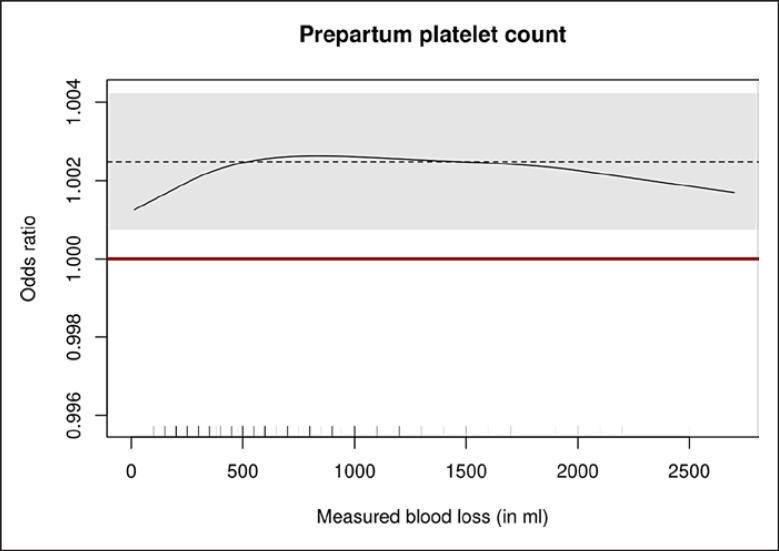
Graph of odds ratios to remain below a certain volume of postpartum blood loss based on measured blood loss (MBL). Dashed line: postpartum blood loss-constant regression coefficient (on the exp-scale) from the continuous outcome logistic regression model; gray area: confidence interval pertaining to the constant regression coefficient; red line: absent effect (odds ratio 1.0); continuous black line: blood loss-cut-off-specific logistic regression coefficients. If the measured blood loss-constant regression coefficient (dashed line) with its confidence interval (gray area) remains above the OR of 1 (red line), this represents a statistically significant influence of increasing platelet numbers on reducing blood loss over the whole spectrum of MBL. The same is true for MBL-specific regression coefficients: here, the effect at any given MBL volume is represented by the continuous line.

According to the graphical representation depicted in Figure 1, the interpretation of constant effects for all MBL values in a continuous outcome logistic regression model can thus be considered appropriate. Using the conventional cut-off of 500 mL MBL (binary logistic regression model, WHO definition for of postpartum hemorrhage), the odds ratio was 1.003 (95% confidence interval, 1.001−1.005, *p* = 0.007).

**Fig. 2:**
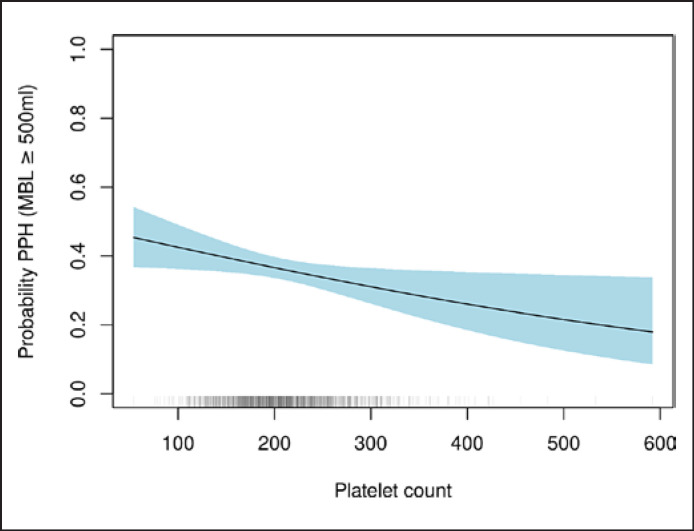
Prevalence of postpartum hemorrhage (PPH) as a function of prepartum platelets for a hypothetical subject with prepartum hemoglobin 127 g/L, prepartum F I 4.5 g/L, prepartum F II 128%, and prepartum F XIII 96% (reflecting the median of the overall population). The blue area represents a 95% confidence band. Rugs on the horizontal axis indicate actually measured prepartum platelet counts; the darkness of a rug reflects the number of measurements of a certain value (the darker the rug, the more frequent this platelet number was observed).

**Fig. 3:**
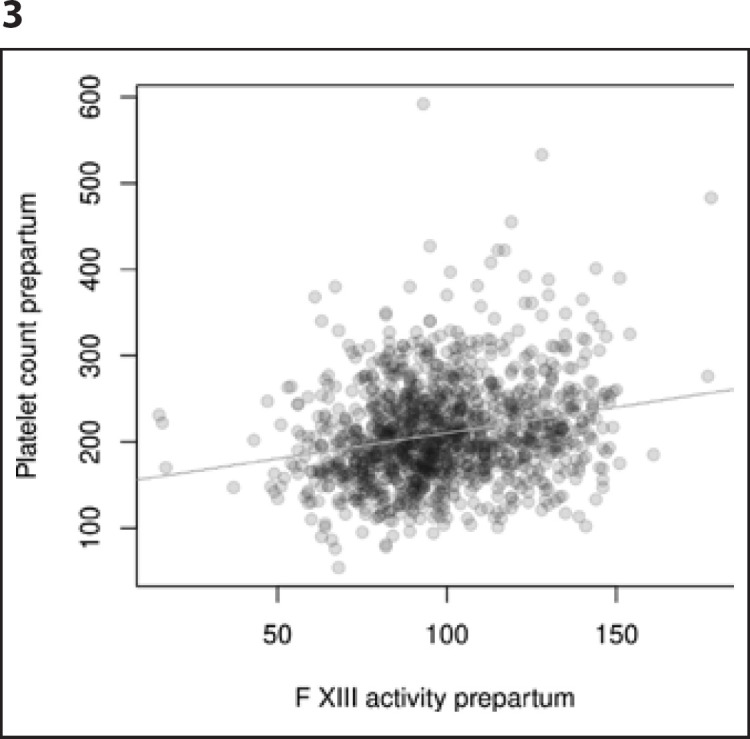
Correlation of prepartum FXIII activity and prepartum platelet count (ƥ 0.228, *p* < 0.001).

**Fig. 4:**
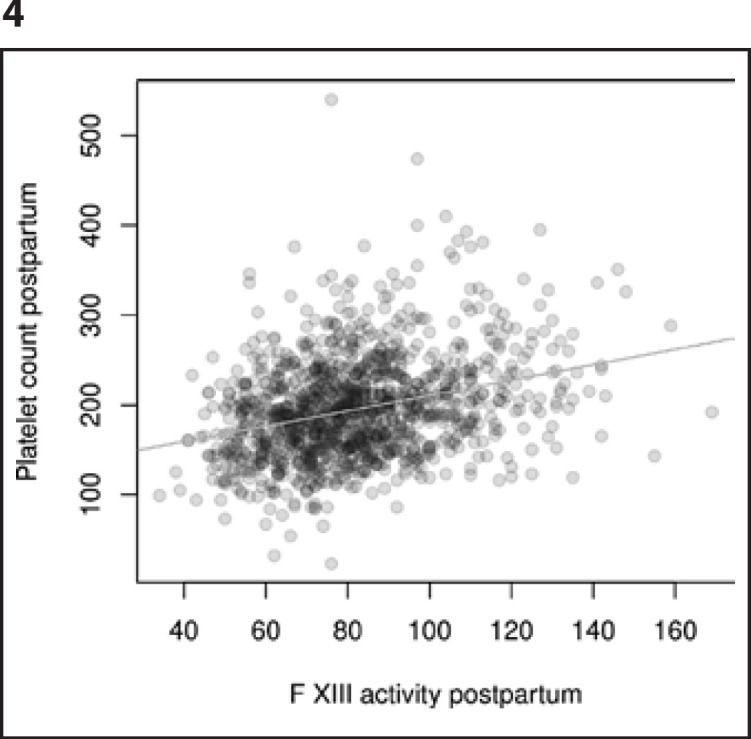
Correlation of postpartum FXIII activity and postpartum blood count (ƥ 0.293, *p* < 0.001).

Having established the association of platelet count with postpartum blood loss, the overall prevalence of postpartum hemorrhage (defined as MBL ≥500 mL) as a function of prepartum platelet count was calculated (Fig. 2). If an option to increase prepartal platelet counts existed, e.g., by 50 G/L, the continuous outcome logistic regression model predicts the probability to remain under any given blood loss volume to rise by 11% and the probability for a PPH to occur to decrease by 16%. In addition to the observations described above, a significant association of prepartum platelet count and prepartum factor XIII activity (Spearman rank correlation coefficient 0.228, *p* < 0.001) as well as postpartum platelet count and postpartum factor XIII activity (Spearman rank correlation coefficient of 0.293, *p* < 0.001) was shown (Fig. 3, 4).

## Discussion

This secondary analysis of a prospective study involving 1,300 parturient women reports important results: that prepartum platelet counts are significantly inversely correlated with the postpartum blood loss, measured with a validated technique and that platelet counts significantly correlate with FXIII activity both pre- and postpartum.

Previous studies have analyzed the association of platelet counts with postpartum blood loss as well, but the blood loss was merely estimated and arbitrarily set thresholds were used (e.g., 500 mL, 1,000 mL, etc., for blood loss and 100 G/L, 150 G/L, etc., for platelet counts). As categorization is prone to a loss of information, this might have led to the contradictory results reported by these studies [2, 3, 4, 5, 6, 7, 8]. In contrast, we performed a continuous outcome logistic regression analysis involving blood coagulation factors I, II, and XIII and analyzed the effect of prepartum platelet counts on postpartum blood loss over the whole spectrum of MBL volumes.

To our knowledge, this study is to date the largest study of postpartum blood loss in parturient women involving blood coagulation factors as well as prepartum platelet count and hemoglobin levels as possible confounders in the analysis of prepartum platelet counts. Our results reveal that every one unit (G/L) increase in prepartum platelet count is associated with an odds ratio of 1.002 to remain below any MBL volume and an odds ratio of 1.003 not to suffer from PPH (MBL >500 mL). To illustrate in other words: an increase of prepartal platelets by 50 G/L rises the probability to remain under any given blood loss volume by 11% and decreases the probability for a PPH to occur by 16%.

The prediction from the continuous outcome logistic regression model that increasing prepartum platelet counts will be associated with a decrease in postpartum blood loss and the frequency of PPH raises the question whether raising the prepartum platelet count could be a novel treatment approach for PPH prevention, albeit the response expected might be smaller than the one expected through FXIII supplementation [12]. The important message, however, is that the biological signal is clearly seen across the entire study population and for all selected blood loss volumes.

This underlines the importance of platelet count in the risk stratification for PPH and the potential use of platelets in the therapy of postpartum hemorrhage. We hence welcome the increased attention that the role of platelets receives in the new German, Austrian, and Swiss guideline [9] for the treatment of PPH.

The American College of Obstetrics and Gynecology suggests in their 2017 practice bulletin that massive transfusion in PPH should be performed according to a protocol with fixed ratios of packed red blood cells, fresh frozen plasma, and platelets. A 1:1:1 ratio was used in over 80% of all American institutions [19]; it remains to be confirmed whether this is the optimal approach as compared to other (ratio of 4:4:1 or 6:4:1) recommendations [20]. The 2007/2008 RCOG guidelines presented in the Green Top Guideline Nr. 47 recommends platelet transfusion in case of platelet counts below 50 G/L in active peripartum bleeding; transfusion can also be considered in (decreasing) platelet counts below 75 G/L to provide a margin of safety [21].

Besides the obvious and established role of platelets in the formation of blood clots, the second finding, the significant association of platelet counts with FXIII activity, both pre- and postpartum, might add valuable information to the importance of platelets on clot firmness. The influence of prepartum FXIII levels on postpartum blood loss has now been proven by our working group [12] and by others [13], while the importance of FXIII has also been recognized in other emergency situations and medical fields [22, 23, 24, 25, 26]. As it has been shown that factor XIII is present in the cytoplasm of platelets [14, 27], it might be postulated that the increased risk for postpartum blood loss not only derives from the reduced absolute number of platelets and the associated reduction in adhesion and aggregation but also from the reduced factor XIII availability at the scene of the platelet effect.

Our report comes with all the usual caveats of a post hoc analysis. However, the greatest strength of our study is the robust prospectively collected data set from a large study population of 1,300 parturient women. The continuous regression analysis performed with these data, including not only prepartum platelet counts but also coagulation factors I, II, and XIII as well as prepartum hemoglobin is, to the best of our knowledge, unique. It is also important to mention that the method we used for blood loss measurement has been clinically validated [15] and thus provides more accurate results than a mere estimation of blood loss volume (a method that has frequently been utilized in other studies).

Our data help explain why even mild thrombocytopenia might increase postpartum blood loss [3, 5]. It might also help clarify why conflicting results have been reported in the past. It seems that increased postpartum blood loss has a multifaceted pathophysiology.

The treatment of PPH must follow a multimodal approach taking into account multiple factors, thrombocytes being one of these. Reduced platelet counts and, potentially, therefore, reduced platelet-derived FXIII activity, in addition to other factors, seem to contribute to increased postpartum blood loss.

We do not assume that many women have severe thrombocytopenia or that only severe thrombocytopenia leads to severe PPH; however, we think that thrombocytopenia might deteriorate blood loss due to another pathology − with the extent of deterioration depending on the number of thrombocytes.

In conclusion, we suggest here that reduced prepartum platelet counts and thus platelet-derived FXIII activity are risk factors for increased postpartum blood loss. We thus support the new German, Austrian, and Swiss guideline on postpartum hemorrhage, which now also underlines the importance of platelet counts and potential platelet transfusions. The association of platelet counts and measured FXIII activity in the peripartal setting requires further investigation.

## Statement of Ethics

All research was performed in accordance with the Declaration of Helsinki. The clinical protocol was reviewed and approved by the Cantonal Ethics Committee Zurich, Switzerland (Institutional Review Board approval (KEK-ZH 2015-0011, ClinicalTrials.gov registration NCT02604602). Informed consent was obtained from all study participants before their study involvement.

## Conflict of Interest Statement

The Center for Laboratory Medicine (Z.L.M.) St. Gallen holds patents on the point of care diagnostics of factor XIII. One of the authors of this manuscript (W.K.) is one of the authors of the new guideline.

## Funding Sources

The initial study was supported by CSL Behring, University Hospital Zurich, the Center for Laboratory Medicine (ZLM) St. Gallen, and a private donor.

## Author Contributions

The initial study outline of the mother study was designed by R. Zimmermann. This secondary analysis of the mother study was designed by R.Brun, C.Haslinger, and W.Korte. C.Haslinger, R.Brun, E.Eigenmann, and M.-L.Frevert collected data and performed its quality control. W.Korte performed the analysis of the coagulation factors. T.Hothorn performed the statistical analysis. R.Brun, W.Korte, and C.Haslinger wrote the first draft of the manuscript. E.Eigenmann, T.Hothorn, M.-L.Frevertm and R.Zimmermann drafted and reviewed the manuscript and contributed to its intellectual content. The final version of the manuscript was approved by all authors prior to publication.

## Data Availability Statement

The data that support the findings of this study are openly available in [repository name “CRAN”] at https://CRAN.R-project.org/package=TH.data.

## Figures and Tables

**Table 1:** Fetomaternal and perinatal characteristics, prepartum hemoglobin, prepartum platelets, and blood coagulation factors, stratified by mode of delivery

Variable	Vaginal delivery (*N =* 677)	Elective cesarean delivery (*N =* 409)	Unplanned cesarean delivery (*N =* 223)
MBL, mL	350 (300-500); (100-5,700)	500 (400-600); (200-2,100)	500(400-700); (200-1,500)
Prepartum hemoglobin, g/L	128.0 (121.0-135.0); (82-1,590)	124.0(118.0-131.0); (96-165)	127.0(120.0-134.0); (89-153)
Prepartum platelet count, G/L	204.0 (172.0-241.0); (86.0-592.0)	205.0 (169.0-244.0); (78.0-442.0)	196.0 (170.0-235.0); 54.0-483.0)
Postpartum platelet count, G/L	196.0 (160.0-231.0); (86.0-540.0)	190.0 (155.0-225.0); (32.0-410.0)	176.0 (149.5-215.0); (23.0-395.0)
Prepartum fibrinogen, g/L	4.5 (3.9-5.1); (0.3-8.5)	4.3 (3.9-4.8); (0.9-8.2)	4.5 (3.9-5.2); (1.6-8.1)
Prepartum factor II activity, %	128.0 (118.0-140.0); (60-223)	128.0(115.0-138.0); (56-184)	128.0(115.0-140.0); (55-185)
Prepartum factor XIII activity, %	98.5 (86.0-117.8); (15.0-177.0)	93.0 (82.0-107.0); (43.0-148.0)	93.0 (80.2-111.0); 37.0-178.0)
Postpartum factor XIII activity, %	84.0(71.0-95.8); (71.0-95.8)	78(67.0-88.0); (38.0-132.0)	72.5 (63.0-82.0); (41.0-155.0)
Number of colloids	0 (0-0); (0.0-4.0)	1 (1-1); (0.0-2.0)	1 (0-1); (0.0-2.0)
Spontaneous delivery	566	−	−
Vacuum delivery	111	−	−
Unplanned cesarean delivery (non-urgent)	−	−	210
Unplanned cesarean delivery (emergency)	−	−	13
Gestational age, days	280 (273-285); (192.0-297.0)	267 (265-270); (185.0-291.0)	277 (268-284); (177.0-294.0)
Maternal age, days	32(29-35); (18.0-45.0)	34 (30-37); (19.0-48.0)	33 (30-36); (19.0-45.0)
Multiparity	310(45.8%)	254 (62.1 %)	70 (31.4%)
Body mass index, kg/m^2^	23.2(20.5-26.8); (15.4-54.5)	25.1 (21.7-28.8); (16.6-66.0)	23.4 (21.0-26.4); (16.4-43.0)
Duration of second stage labor, minutes	51 (18-121); (2.0-344.0)	−	173(119-207); (0.0-258.0)
Multiple fetus pregnancy	6 (0.9%)	32 (7.8%)	13 (5.8%)
Induction of labor	263 (38.8%)	4(1.0%)	85 (38.1%)
Induction of labor >48 h	22 (3.2%)	1 (0.2%)	18(8.1%)
Chorioamnionitis	1 (0.1%)	0	9 (4.0%)
Neonatal weight, g	3,370 (3,090-3,650); (990.0-4,570.0)	3,200 (2,890-3,510); (360.0-4,550.0)	3,340 (2,945-3,690); (800.0-4,630.0)
Uterine rupture	0	0	3(1.3%)
Uterine atony	42 (6.2%)	7(1.7%)	4(1.8%)
Retained placenta	24 (3.5%)	0	0
Retained placental tissue	26 (3.8%)	1 (0.2%)	0
Morbidly adherent placenta	1 (0.1%)	3 (0.7%)	1 (0.4%)
Placenta previa	0	9 (2.2%)	4(1.8%)
Bleeding from laceration	49 (7.2%)	0	0
Placental abruption	2 (0.3%)	2 (4.9%)	6 (2.7%)

Data are median (interquartile range); (range) or *n* (%).
